# Cross-national time trends in adolescent alcohol use from 2002 to 2014

**DOI:** 10.1093/eurpub/ckab024

**Published:** 2021-07-14

**Authors:** Eva Leal-López, Inmaculada Sánchez-Queija, Alessio Vieno, Dorothy Currie, , Torbjorn Torsheim, Daria Pavlova, Concepción Moreno-Maldonado, , Bart De Clercq, Michal Kalman, Joanna Inchley

**Affiliations:** Department of Developmental and Educational Psychology, Universidad de Sevilla, Seville, Spain; Department of Developmental and Educational Psychology, Universidad de Sevilla, Seville, Spain; Department of Developmental and Social Psychology, University of Padova, Padova, Italy; School of Medicine, University of St Andrews, St Andrews, UK; Department of Psychosocial Science, University of Bergen, Bergen, Norway; Ukrainian Institute for Social Research After Olexander Yaremenko, Kyiv, Ukraine; Department of Developmental and Educational Psychology, Universidad de Sevilla, Seville, Spain; Mensura R&D Department, Mensura EDPB, Antwerpen, Belgium; Department of Recreation and Leisure Studies, Palacky University Olomouc, Olomouc, Czech Republic; MRC/CSO Social and Public Health Sciences Unit, University of Glasgow, Glasgow, UK

## Abstract

**Background:**

Adolescent alcohol consumption is a major public health concern that should be continuously monitored. This study aims (i) to analyze country-level trends in weekly alcohol consumption, drunkenness and early initiation in alcohol consumption and drunkenness among 15-year-old adolescents from 39 countries and regions across Europe and North America between 2002 and 2014 and (ii) to examine the geographical patterns in adolescent alcohol-related behaviours.

**Methods:**

The sample was composed of 250 161 adolescents aged 15 from 39 countries and regions from Europe and North America. Survey years were 2002, 2006, 2010 and 2014. The alcohol consumption and drunkenness items of the HBSC questionnaire were employed. Prevalence ratios and 95% confidence intervals were estimated using Poisson regression models with robust variance.

**Results:**

Data show a general decrease in all four alcohol variables between 2002 and 2014 except for some countries. However, there is variability both within a country (depending on the alcohol-related behaviour under study) and across countries (in the beginning and shape of trends). Some countries have not reduced or even increased their levels in some variables. Although some particularities have persisted over time, there are no robust patterns by regions.

**Conclusions:**

Despite an overall decrease in adolescent alcohol consumption, special attention should be paid to those countries where declines are not present, or despite decreasing, rates are still high. Further research is needed to clarify factors associated with adolescent drinking, to better understand country specificities and to implement effective policies.

## Introduction 

Adolescent alcohol consumption has declined for the last two decades in many countries.^1–4^ In Europe, ESPAD data showed an overall decrease in lifetime alcohol use from 90% in 2003 to 81% in 2015.^1^ However, trends vary between countries, whereby decreases combine with periods of stability and even increases. In a recent study on past-month drinking, Vashishtha et al.^5^ found that results varied markedly across countries, with Northern countries showing the earliest and steepest declines (the British Isles countries also showed large reductions), whereas the Eastern and Southern countries showed the shallowest declines. The variability in trends between countries suggests important socio-cultural influences on adolescent drinking and highlights the importance of ongoing monitoring of alcohol-related behaviours among this age group in order to (i) better understand how behaviours change over time, (ii) identify what may be influencing such trends and (iii) inform evidence-based public health policies.

Within Europe, there is not a strong consensus on geographical drinking patterns. Traditionally, most studies found more frequent but moderate drinking in Southern countries, while less regular but heavier drinking in Northern countries.^6^ In a recent analysis with ESPAD data, this pattern was supported, showing Western and Southern Europe higher weekly use compared with Northern Europe.^7^ A different categorization of countries [‘mainly non-using’, ‘mainly mild but frequent’ and ‘highest proportions of (heavy) episodic drinking’] was also proposed.^8^ Other studies, however, suggested a cultural convergence across countries.^9–11^ Therefore, it seems that current geographical patterns in adolescent alcohol consumption are unclear, and traditional patterns may have changed over time.

We have a unique opportunity to address these gaps in knowledge through the data from the cross-national *Health Behaviour in School-aged Children* (HBSC) study. In this article, we expand the single-measure approach and the regional analysis and explore four different alcohol-related behaviours in each country to have a broader perspective of what is happening with adolescent drinking and how countries group by data. In particular, the aims of this study are (i) to analyze country-level trends in weekly alcohol consumption, drunkenness and early initiation in alcohol consumption and drunkenness among 15-year-old adolescents from 39 countries/regions across Europe and North-America between 2002 and 2014 and (ii) to examine geographical patterns in the four adolescent alcohol-related behaviours.

## Methods

### Participants

The HBSC study is a cross-national study of adolescent health and wellbeing conducted every 4 years in association with the World Health Organization in more than 50 European and North American countries and regions. This school-based study uses a self-report survey administered to whole school classes. Data from each country is collected according to the HBSC international protocol for each survey round to ensure consistency in survey instruments, data collection and processing procedures.^12^ Samples are designed to be nationally representative of pupils aged 11, 13 and 15 years. Data for this article is based on the age group with higher alcohol consumption, 15-year-old pupils (e.g. in 2014, 3% of 11-year-olds and 5% of 13-year-olds reported weekly drinking compared with 13% at age 15^4^). We used HBSC data collected from 39 countries/regions that participated in at least three survey waves conducted in 2002, 2006, 2010 and 2014. Data from the 2018 survey could not be included because no comparable data was available for three of the four measures, and the focus of this work is on comparing different patterns of alcohol consumption in the same period of time both within and across countries. The sample size was 250 161 adolescents, mean age 15.54 years (51.3% girls) (see Supplementary Material A).

### Measures

#### Weekly alcohol use.

Weekly alcohol use was evaluated with the question ‘At present, how often do you drink anything alcoholic? Try to include even those times when you only drink a small amount’. The items were beer, wine and spirits. For each item, response options were ‘1 = every day’, ‘2 = every week’, ‘3 = every month’, ‘4 = rarely’ and ‘5 = never’. An overall alcohol use index was created considering the highest frequency of any alcoholic beverage consumed. This variable was dichotomized into ‘weekly alcohol use’ (options 1 and 2) and ‘less than weekly’ (options 3, 4 and 5).

#### Drunkenness

Drunkenness in a lifetime was assessed with the question ‘Have you ever had so much alcohol that you were really drunk?’. Response options were ‘1 = never’, ‘2 = once’, ‘3 = 2–3 times’, ‘4 = 4–10 times’ and ‘5 = more than 10 times’. This variable was dichotomized into ‘never or once’ (options 1 and 2) and ‘two times or more’ (options 3, 4 and 5).

#### Early initiation (at age 13 or younger) in alcohol use and drunkenness.

Adolescents were asked at what age they had drunk alcohol for the first time and had been drunk for the first time. Response options ranged from 11 years old or younger to 16 years old or older. ‘Early initiation’ was classified as age 13 or younger.^1^

### Statistical analyses

Poisson regressions models with robust variance were used to analyze trends for the global comparison (2002–14) and partial comparisons (2002–06, 2006–10 and 2010–14). In all comparisons, the first year served as the reference category. For Iceland, Luxembourg, Romania and Slovakia, the global comparison refers to 2006–14 (no data in 2002). Models were run for each country separately. Prevalence ratios (PRs) with 95% confidence intervals were calculated. All analyses incorporated post-stratification weights were provided. Data analyses were conducted using STATA/SE 12.^13^

## Results

### Weekly alcohol use

Weekly drinking decreased between 2002 and 2014 in all countries except Israel, Republic of North Macedonia and Romania (table 1). Most countries started the decline in 2002, but only nine (Belgium Flemish, Belgium French, Canada, Denmark, England, Estonia, Netherlands, Scotland and Wales) showed a linear trend. In six countries, the decline began in 2006 while in nine countries in 2010. Two-thirds of the countries reduced their weekly drinking between 2002 and 2014 by around half, with greatest reduction (∼70%) observed in countries showing highest percentages in 2002, such as Denmark, England, Scotland, Ukraine and Wales as well as in other countries with lower percentages in 2002 (Estonia, Greenland, Iceland, Ireland, Latvia, Norway, Russian Federation, Sweden and Switzerland). In 2014, the highest values (>20%) were observed in Malta, Croatia, Italy, Hungary and Greece, whereas the lowest ones (5% or less) were found in Iceland, Norway, Sweden, Ireland, Greenland, Finland, Latvia and Estonia.

**Table 1:** Weekly alcohol use among adolescents aged 15 by country: percentage in each survey year, percentage of change between 2002^a^ and 2014, and PR (95% CI) of global comparison (2002–14)^a^ and 4-year period comparisons (2002–06, 2006–10 and 2010–14)

Country	2002 (%)	2006 (%)	2010 (%)	2014 (%)	Change 2002–14^a^ (%)	Global comparison (2002–14)^a^ PR (95% CI)	2002–06 PR (95% CI)	2006–10 PR (95% CI)	2010–14 PR (95% CI)
Austria	25.2	30.7	27.6	14.2	−44	0.565 (0.479–0.667)	1.219 (1.079–1.377)	0.900 (0.809–1.001)	0.515 (0.441–0.602)
Belgium (Flemish)	30.6	27.1	22.6	13.6	−56	0.444 (0.386–0.509)	0.886 (0.799–0.983)	0.833 (0.731–0.950)	0.602 (0.514–0.706)
Belgium (French)	24.9	21.3	17.5	11.2	−55	0.450 (0.385–0.527)	0.857 (0.747–0.982)	0.824 (0.706–0.962)	0.637 (0.536–0.757)
Canada	22.1	15.0	12.6	9.2	−59	0.416 (0.362–0.478)	0.680 (0.588–0.786)	0.842 (0.746–0.950)	0.728 (0.650–0.815)
Croatia	26.5	31.8	30.1	21.5	−19	0.810 (0.717–0.915)	1.201 (1.074–1.344)	0.945 (0.861–1.038)	0.713 (0.642–0.793)
Czech Republic	34.7	32.6	36.5	15.0	−57	0.433 (0.380–0.493)	0.939 (0.853–1.033)	1.120 (1.017–1.233)	0.412 (0.362–0.469)
Denmark	44.6	29.0	17.5	11.6	−74	0.260 (0.221–0.307)	0.649 (0.588–0.716)	0.604 (0.522–0.699)	0.665 (0.547–0.808)
England	46.4	32.4	16.2	8.2	−82	0.177 (0.149–0.211)	0.699 (0.639–0.765)	0.499 (0.428–0.582)	0.509 (0.412–0.630)
Estonia	19.9	16.4	12.0	5.3	−73	0.266 (0.205–0.344)	0.823 (0.704–0.963)	0.730 (0.609–0.875)	0.442 (0.336–0.581)
Finland	10.5	8.4	5.4	4.6	−56	0.434 (0.340–0.556)	0.800 (0.649–0.986)	0.647 (0.510–0.822)	0.840 (0.640–1.102)
France	14.9	14.6	14.4	9.4	−37	0.635 (0.535–0.755)	0.983 (0.857–1.126)	0.986 (0.850–1.145)	0.655 (0.546–0.787)
Germany	29.4	17.1	16.0	10.8	−63	0.367 (0.318–0.423)	0.582 (0.520–0.652)	0.934 (0.812–1.075)	0.674 (0.571–0.797)
Greece	30.1	26.7	32.3	20.5	−32	0.680 (0.595–0.779)	0.886 (0.787–0.999)	1.210 (1.082–1.352)	0.634 (0.559–0.721)
Greenland	13.2	5.7	5.6	4.2	−68	0.318 (0.170–0.594)	0.435 (0.260–0.728)	0.968 (0.545–1.721)	0.755 (0.384–1.484)
Hungary	28.8	24.2	22.5	20.6	−29	0.716 (0.620–0.827)	0.840 (0.736–0.958)	0.931 (0.814–1.065)	0.916 (0.792–1.059)
Iceland	–	11.1	5.7	2.5	−77	0.226 (0.176–0.266)	–	0.514 (0.427–0.618)	0.439 (0.342–0.565)
Ireland	12.4	14.7	9.3	3.1	−75	0.249 (0.178–0.348)	1.183 (0.958–1.463)	0.638 (0.528–0.771)	0.330 (0.240–0.455)
Israel	16.7	11.6	18.1	16.8	0	1.005 (0.860–1.176)	0.695 (0.586–0.823)	1.554 (1.309–1.845)	0.932 (0.795–1.092)
Italy	37.0	33.7	24.4	20.9	−44	0.565 (0.496–0.643)	0.912 (0.821–1.014)	0.724 (0.645–0.813)	0.855 (0.744–0.983)
Latvia	15.3	18.3	15.4	4.7	−69	0.307 (0.238–0.396)	1.194 (0.998–1.428)	0.838 (0.708–0.992)	0.307 (0.240–0.393)
Lithuania	20.3	13.0	15.7	7.7	−62	0.378 (0.313–0.457)	0.638 (0.550–0.740)	1.207 (1.028–1.417)	0.492 (0.403–0.599)
Luxembourg	–	17.5	16.9	9.0	−49	0.514 (0.412–0.641)	–	0.962 (0.819–1.131)	0.534 (0.427–0.668)
Malta	47.3	42.3	–	26.6	−44	0.561 (0.482–0.654)	0.894 (0.772–1.036)	–	–
Netherlands	33.9	26.6	17.9	12.3	−64	0.361 (0.307–0.425)	0.785 (0.698–0.883)	0.671 (0.582–0.773)	0.685 (0.572–0.821)
Norway	16.5	9.0	7.7	3.0	−82	0.184 (0.125–0.271)	0.544 (0.447–0.661)	0.856 (0.666–1.101)	0.396 (0.260–0.601)
Poland	13.2	10.7	12.1	9.5	−28	0.721 (0.596–0.873)	0.809 (0.688–0.950)	1.134 (0.943–1.363)	0.787 (0.637–0.972)
Portugal	16.0	10.8	7.8	6.8	−58	0.424 (0.329–0.548)	0.675 (0.542–0.842)	0.721 (0.573–0.907)	0.872 (0.670–1.134)
Rep. North Macedonia	13.9	18.3	14.7	12.9	−1	0.933 (0.774–1.124)	1.322 (1.124–1.554)	0.805 (0.690–0.938)	0.877 (0.733–1.050)
Romania	–	14.6	18.7	15.3	+1	1.049 (0.885–1.244)	–	1.285 (1.105–1.493)	0.817 (0.701–0.952)
Russian Federation	24.3	18.3	8.6	6.8	−72	0.281 (0.229–0.345)	0.755 (0.680–0.839)	0.469 (0.395–0.557)	0.793 (0.620–1.014)
Scotland	40.8	28.1	20.8	9.9	−76	0.243 (0.208–0.283)	0.689 (0.625–0.759)	0.741 (0.670–0.820)	0.475 (0.406–0.556)
Slovakia	–	20.4	19.3	14.2	–30	0.695 (0.594–0.814)	–	0.947 (0.821–1.093)	0.734 (0.635–0.849)
Slovenia	26.5	24.7	25.5	11.9	−55	0.447 (0.378–0.528)	0.932 (0.816–1.065)	1.030 (0.916–1.158)	0.465 (0.399–0.543)
Spain	20.9	–	20.6	8.3	−60	0.395 (0.344–0.455)	–	–	0.402 (0.350–0.461)
Sweden	15.2	7.3	6.6	3.1	−80	0.206 (0.161–0.264)	0.479 (0.383–0.600)	0.914 (0.718–1.165)	0.470 (0.361–0.612)
Switzerland	23.9	17.3	17.9	7.8	−67	0.326 (0.275–0.387)	0.725 (0.628–0.837)	1.033 (0.895–1.191)	0.436 (0.368–0.516)
Ukraine	30.6	46.2	30.5	10.2	−67	0.332 (0.283–0.390)	1.510 (1.381–1.651)	0.688 (0.638–0.743)	0.333 (0.284–0.390)
USA	14.2	10.5	8.6	–	–	–	0.719 (0.588–0.879)	0.818 (0.657–1.019)	–
Wales	39.7	30.4	24.2	10.5	−74	0.265 (0.224–0.313)	0.766 (0.687–0.853)	0.798 (0.709–0.898)	0.434 (0.364–0.516)

a For Iceland, Luxembourg, Romania, and Slovakia the change and the global comparison refer to 2006-2014.

### Drunkenness

Prevalence of drunkenness (two times or more in a lifetime) also declined in most countries between 2002 and 2014 (table 2). However, stability in the global comparison was observed in five countries (Czech Republic, France, Greece, Hungary and the Republic of North Macedonia) and an overall increase was detected in two (Croatia and Malta). Declines were evident from 2002 in 15 countries, but only five showed a linear downward trend from 2002 to 2014 (England, Finland, Ukraine, USA and Wales), whereas the others showed a period of stability or increase in 2006–10. In the remaining countries, the decline began in 2006 (10 countries) or 2010 (11 countries). Decreases by around half between 2002 and 2014 were found in nine countries (England, Greenland, Iceland, Ireland, Norway, Russian Federation, Sweden, Switzerland and Ukraine). In 2014, percentages above 30% were found in Denmark, Hungary, Lithuania, Wales, Croatia, Scotland, Slovenia, Estonia and the Czech Republic. On the contrary, Iceland, Israel, Republic of North Macedonia, Switzerland, Russian Federation and Luxembourg presented percentages below 15%.

**Table 2:** Been drunk two times or more in lifetime among adolescents aged 15 by country: percentage in each survey year, percentage of change between 2002^a^ and 2014, and PR (95% CI) of global comparison (2002–14)^a^ and 4-year period comparisons (2002–06, 2006–10 and 2010–14)

Country	2002 (%)	2006 (%)	2010 (%)	2014 (%)	Change 2002–14^a^ (%)	Global comparison (2002–14)^a^ PR (95% CI)	2002–06 PR (95% CI)	2006–10 PR (95% CI)	2010–14 PR (95% CI)
Austria	36.4	38.5	35.2	22.9	−37	0.630 (0.559–0.714)	1.056 (0.958–1.163)	0.915 (0.837–1.001)	0.652 (0.579–0.735)
Belgium (Flemish)	31.8	28.4	28	21.1	−34	0.664 (0.593–0.742)	0.893 (0.807–0.987)	0.986 (0.875–1.110)	0.754 (0.663–0.857)
Belgium (French)	27.5	26.3	24.5	20.3	−26	0.738 (0.652–0.835)	0.954 (0.844–1.079)	0.932 (0.819–1.060)	0.830 (0.730–0.945)
Canada	42.3	35.3	34.0	23.2	−45	0.522 (0.471–0.579)	0.835 (0.765–0.910)	0.967 (0.896–1.043)	0.647 (0.589–0.712)
Croatia	28.4	37.8	34.8	32.3	+14	1.138 (1.024–1.264)	1.333 (1.202–1.478)	0.919 (0.846–0.998)	0.929 (0.853–1.012)
Czech Republic	33.1	33.5	43.0	30.0	−9	0.906 (0.821–1.001)	1.010 (0.918–1.113)	1.283 (1.173–1.403)	0.699 (0.638–0.767)
Denmark	66.1	57.3	55.3	38.2	−42	0.578 (0.534–0.626)	0.867 (0.818–0.918)	0.966 (0.903–1.032)	0.691 (0.634–0.753)
England	54.9	46.8	35.3	28.1	−49	0.511 (0.467–0.559)	0.852 (0.795–0.913)	0.872 (0.792–0.959)	0.689 (0.616–0.770)
Estonia	49.2	49.7	44.8	30.1	−39	0.612 (0.553–0.677)	1.010 (0.937–1.089)	0.901 (0.835–0.973)	0.673 (0.607–0.745)
Finland	54.5	45.4	40.7	29.7	−46	0.544 (0.502–0.590)	0.832 (0.778–0.891)	0.896 (0.832–0.964)	0.730 (0.670–0.796)
France	18.6	23.5	21.4	18.1	−3	0.891 (0.777–1.022)	1.265 (1.133–1.412)	0.910 (0.811–1.020)	0.775 (0.673–0.892)
Germany	39.2	29.4	30.7	24.4	−38	0.624 (0.567–0.687)	0.751 (0.690–0.817)	1.042 (0.947–1.145)	0.798 (0.719–0.887)
Greece	20.1	19.2	22.4	21.2	+5	1.057 (0.910–1.228)	0.854 (0.818–1.113)	1.173 (1.018–1.352)	0.945 (0.823–1.084)
Greenland	57.6	43.9	46.9	24.2	−58	0.420 (0.334–0.529)	0.762 (0.653–0.889)	1.069 (0.918–1.245)	0.516 (0.411–0.649)
Hungary	34.3	35.7	39.9	37.2	+9	1.087 (0.976–1.211)	1.043 (0.937–1.161)	1.138 (1.034–1.254)	0.915 (0.831–1.008)
Iceland	–	31.7	17.1	6.0	−81	0.188 (0.162–0.219)	–	0.540 (0.490–0.595)	0.349 (0.299–0.407)
Ireland	32.0	33.6	29.0	16.3	−49	0.508 (0.438–0.589)	1.050 (0.935–1.179)	0.864 (0.781–0.955)	0.560 (0.489–0.642)
Israel	15.2	12.6	15.5	9.1	−40	0.656 (0.538–0.800)	0.996 (0.838–1.185)	1.019 (0.852–1.218)	0.646 (0.527–0.792)
Italy	19.1	20.1	16.4	16.0	−16	0.840 (0.708–0.997)	1.051 (0.897–1.230)	0.817 (0.699–0.954)	0.979 (0.827–1.160)
Latvia	32.4	44.4	46.4	27.8	−14	0.859 (0.767–0.963)	1.372 (1.237–1.523)	1.045 (0.962–1.136)	0.599 (0.545–0.659)
Lithuania	49.7	53.6	52.4	36.9	−26	0.743 (0.688–0.803)	1.077 (1.013–1.146)	0.979 (0.921–1.041)	0.705 (0.653–0.760)
Luxembourg	–	23.7	18.3	14.3	−40	0.603 (0.508–0.717)	–	0.771 (0.667–0.891)	0.783 (0.651–0.942)
Malta	20.8	16.8	–	27.1	+30	1.305 (1.071–1.589)	0.806 (0.609–1.065)	–	–
Netherlands	28.4	25.7	18.4	16.5	−42	0.585 (0.502–0.681)	0.905 (0.798–1.027)	0.704 (0.610–0.814)	0.917 (0.775–1.084)
Norway	39.5	28.3	26.6	19.2	−51	0.485 (0.419–0.562)	0.715 (0.647–0.790)	0.942 (0.836–1.062)	0.720 (0.613–0.846)
Poland	30.9	34.4	30.6	25.8	−17	0.835 (0.750–0.930)	1.111 (1.020–1.210)	0.891 (0.809–0.982)	0.843 (0.750–0.948)
Portugal	22.1	21.0	20.5	16.5	−25	0.746 (0.624–0.892)	0.954 (0.808–1.126)	0.976 (0.847–1.125)	0.801 (0.686–0.937)
Rep. North Macedonia	11.1	18.4	13.6	11.6	+5	1.044 (0.850–1.283)	1.658 (1.390–1.978)	0.742 (0.633–0.868)	0.849 (0.702–1.028)
Romania	–	28.7	33.1	19.6	−32	0.684 (0.601–0.780)	–	1.153 (1.043–1.275)	0.594 (0.525–0.671)
Russian Federation	33.7	34.5	21.6	14.1	−59	0.418 (0.363–0.481)	1.025 (0.950–1.104)	0.627 (0.565–0.694)	0.651 (0.556–0.761)
Scotland	51.8	45.1	43.2	31.6	−39	0.630 (0.574–0.691)	0.870 (0.809–0.935)	0.958 (0.898–1.021)	0.757 (0.694–0.825)
Slovakia	–	35.2	35.1	27.6	−22	0.784 (0.705–0.872)	–	0.998 (0.905–1.099)	0.786 (0.714–0.865)
Slovenia	39.2	35.2	40.7	30.3	−23	0.772 (0.694–0.858)	0.898 (0.811–0.993)	1.156 (1.059–1.262)	0.744 (0.678–0.816)
Spain	25.3	–	33.8	20.8	−18	0.823 (0.743–0.911)	–	–	0.616 (0.564–0.673)
Sweden	38.6	26.1	24.0	16.4	−58	0.426 (0.381–0.475)	0.676 (0.605–0.755)	0.920 (0.820–1.031)	0.685 (0.611–0.768)
Switzerland	32.3	23.1	23.5	13.7	−58	0.423 (0.372–0.481)	0.717 (0.637–0.806)	1.014 (0.900–1.143)	0.583 (0.512–0.663)
Ukraine	51.7	35.1	30.3	17.7	−66	0.335 (0.299–0.375)	0.660 (0.610–0.715)	0.869 (0.790–0.954)	0.584 (0.516–0.661)
USA	26.3	19.8	14.1	–	–	–	0.754 (0.657–0.866)	0.713 (0.608–0.835)	–
Wales	59.2	53.3	48.5	34.4	−42	0.522 (0.472–0.578)	0.899 (0.839–0.964)	0.910 (0.848–0.977)	0.638 (0.576–0.707)

a For Iceland, Luxembourg, Romania and Slovakia the change and the global comparison refer to 2006–14.

### Early onset in alcohol use

The proportion of adolescents who first drank alcohol at age 13 or younger (table 3) decreased in all countries except for Slovenia (increase) and Croatia, Estonia, Greece and Italy (stability). One-quarter of countries showed a linear downward trend since 2002, and four more countries decreased from 2002 to 2014 but remained stable between 2006 and 2010. Likewise, about a quarter showed a linear decreasing pattern since 2006, and three more countries decreased from 2006 to 2014 but remained stable between 2010 and 2014. A small group of countries started to decline in 2010 (among them, those not decreasing in the global comparison). Reductions by around half between 2002 and 2014 were found in more than one-third of countries, with steepest declines (more than 60%) in Belgium French, Czech Republic, Iceland, Norway, Sweden and Wales. In 2014, in Estonia, Lithuania, Greece, Hungary and Croatia, more than 40% of the 15-year-olds drank at age 13 or earlier, whereas in Iceland, Israel, Sweden and Norway, the percentages were below 15%.

**Table 3:** Early initiation in alcohol consumption (13 years old or younger) among adolescents aged 15 by country: percentage in each survey year, percentage of change between 2002^a^ and 2014, and PR (95% CI) of global comparison (2002–14)^a^ and 4-year period comparisons (2002–06, 2006–10 and 2010–14)

Country	2002 (%)	2006 (%)	2010 (%)	2014 (%)	Change 2002–14^a^ (%)	Global comparison (2002–14)^a^ PR (95% CI)	2002–06 PR (95% CI)	2006–10 PR (95% CI)	2010–14 PR (95% CI)
Austria	70.3	60.4	44.0	39.0	−45	0.555 (0.513–0.600)	0.859 (0.813–0.908)	0.729 (0.681–0.781)	0.886 (0.811–0.967)
Belgium (Flemish)	59.1	53.1	48.0	25.9	−56	0.438 (0.401–0.479)	0.899 (0.848–0.954)	0.903 (0.839–0.973)	0.539 (0.488–0.595)
Belgium (French)	55.4	58.9	40.3	21.4	−61	0.386 (0.350–0.426)	1.063 (0.996–1.135)	0.685 (0.632–0.741)	0.530 (0.476–0.591)
Canada	39.3	35.5	32.9	22.0	−44	0.596 (0.536–0.662)	0.906 (0.828–0.991)	0.932 (0.863–1.007)	0.706 (0.642–0.775)
Croatia	42.0	47.4	50.1	40.0	−5	0.951 (0.875–1.033)	1.127 (1.040–1.221)	1.059 (0.992–1.130)	0.797 (0.744–0.854)
Czech Republic	73.4	68.6	57.8	24.9	−66	0.340 (0.311–0.371)	0.935 (0.894–0.977)	0.843 (0.798–0.890)	0.431 (0.393–0.473)
Denmark	58.9	48.2	44.9	33.3	−43	0.565 (0.516–0.619)	0.817 (0.763–0.876)	0.933 (0.859–1.013)	0.741 (0.669–0.820)
England	56.1	49.6	39.4	29.3	−48	0.526 (0.482–0.574)	0.891 (0.832–0.953)	0.900 (0.823–0.984)	0.656 (0.590–0.729)
Estonia	52.4	58.0	61.5	48.6	−7	0.928 (0.859–1.003)	1.108 (1.035–1.185)	1.060 (0.999–1.125)	0.790 (0.737–0.848)
Finland	50.8	32.0	28.0	21.0	−59	0.413 (0.373–0.457)	0.631 (0.578–0.688)	0.873 (0.791–0.965)	0.749 (0.670–0.838)
Germany	52.3	48.0	43.7	36.5	−30	0.700 (0.650–0.753)	0.918 (0.863–0.976)	0.908 (0.847–0.975)	0.839 (0.774–0.909)
Greece	41.7	46.1	46.1	42.5	+2	1.021 (0.932–1.118)	1.121 (1.027–1.223)	0.986 (0.910–1.069)	0.924 (0.849–1.004)
Hungary	51.9	52.2	46.9	41.3	−20	0.796 (0.729–0.870)	1.005 (0.930–1.086)	0.895 (0.829–0.967)	0.885 (0.810–0.968)
Iceland	–	14.3	11.2	5.3	−63	0.371 (0.309–0.445)	–	0.779 (0.674–0.899)	0.476 (0.401–0.565)
Ireland	39.6	38.2	33.4	16.9	−57	0.427 (0.372–0.490)	0.965 (0.872–1.068)	0.874 (0.797–0.958)	0.506 (0.444–0.578)
Israel	20.5	–	19.0	9.3	−55	0.485 (0.390–0.603)	–	–	0.529 (0.425–0.660)
Italy	21.4	27.1	28.4	19.1	−11	0.892 (0.761–1.046)	1.267 (1.100–1.460)	1.049 (0.928–1.185)	0.672 (0.583–0.774)
Latvia	33.8	49.2	51.4	28.6	−15	0.847 (0.755–0.949)	1.453 (1.311–1.611)	1.046 (0.968–1.131)	0.557 (0.508–0.611)
Lithuania	58.1	54.5	57.1	42.7	−27	0.734 (0.686–0.786)	0.937 (0.885–0.992)	1.048 (0.989–1.111)	0.748 (0.698–0.801)
Luxembourg	–	47.5	31.7	29.3	−38	0.617 (0.554–0.687)	–	0.667 (0.607–0.733)	0.925 (0.819–1.045)
Malta	36.8	39.9	–	25.0	−32	0.679 (0.573–0.804)	1.083 (0.918–1.277)	–	–
Netherlands	52.8	61.9	43.1	26.1	−51	0.502 (0.452–0.559)	1.172 (1.096–1.254)	0.690 (0.640–0.743)	0.622 (0.556–0.695)
Norway	37.7	23.0	18.7	14.6	−61	0.388 (0.326–0.461)	0.610 (0.545–0.683)	0.814 (0.703–0.942)	0.782 (0.644–0.950)
Poland	50.2	53.6	47.7	31.9	−36	0.635 (0.581–0.695)	1.068 (1.008–1.132)	0.890 (0.832–0.952)	0.668 (0.607–0.735)
Portugal	42.1	45.5	41.4	37.7	−10	0.894 (0.803–0.996)	1.079 (0.976–1.194)	0.910 (0.837–0.989)	0.911 (0.831–0.998)
Rep. North Macedonia	30.0	29.7	32.2	23.6	−21	0.787 (0.696–0.891)	0.989 (0.888–1.100)	1.085 (0.980–1.201)	0.734 (0.652–0.826)
Romania	–	33.6	27.3	28.8	−14	0.858 (0.770–0.955)	–	0.813 (0.735–0.901)	1.054 (0.945–1.177)
Russian Federation	30.7	42.7	25.8	16.3	−47	0.530 (0.462–0.607)	1.393 (1.293–1.500)	0.603 (0.548–0.663)	0.631 (0.544–0.732)
Scotland	58.3	48.3	43.6	27.1	−52	0.484 (0.439–0.533)	0.828 (0.775–0.884)	0.903 (0.848–0.960)	0.648 (0.590–0.712)
Slovakia	–	52.1	31.6	25.3	−54	0.486 (0.440–0.536)	–	0.606 (0.555–0.663)	0.801 (0.718–0.893)
Slovenia	29.7	40.7	45.1	39.0	+31	1.314 (1.171–1.474)	1.371 (1.223–1.537)	1.108 (1.023–1.200)	0.865 (0.798–0.937)
Spain	37.2	34.5	42.1	26.3	−29	0.706 (0.645–0.772)	0.920 (0.837–1.011)	1.230 (1.119–1.353)	0.624 (0.569–0.683)
Sweden	38.8	24.3	23.4	14.3	−63	0.369 (0.329–0.415)	0.625 (0.558–0.701)	0.964 (0.856–1.086)	0.613 (0.543–0.691)
Switzerland	43.1	45.1	36.7	25.9	−40	0.600 (0.547–0.659)	1.045 (0.963–1.134)	0.814 (0.752–0.881)	0.706 (0.645–0.773)
Ukraine	38.3	42.6	31.1	30.5	−20	0.791 (0.718–0.872)	1.095 (1.005–1.192)	0.737 (0.675–0.805)	0.980 (0.887–1.083)
Wales	66.7	44.3	40.0	26.5	−60	0.376 (0.335–0.423)	0.665 (0.618–0.715)	0.901 (0.828–0.981)	0.628 (0.555–0.711)

France, Greenland and USA are not included because they participated in only two rounds.

a For Iceland, Luxembourg, Romania and Slovakia, the change and the global comparison refer to 2006–14.

### Early onset in drunkenness

The proportion of adolescents who reported having been drunk at age 13 or younger (table 4) decreased from 2002 to 2014 in all countries except Greece, Hungary, Latvia, Malta and the Republic of North Macedonia, where no change was observed. Again, there was variation in timing; 10 countries started in 2002, 11 in 2006 and 14 in 2010. England, Finland, Norway and Wales were the only countries showing a linear downward trend from 2002 to 2014. More than half of the countries presented reductions by around half between 2002 and 2014, with greatest decreases (∼70%) observed in Austria, Belgium Flemish, Belgium French, Denmark, Finland, Iceland, Ireland, Norway, Russian Federation, Sweden and Wales. In 2014, the proportion of 15-year-olds that had been drunk at age 13 or younger was higher in Lithuania, Estonia, Latvia, Finland, Croatia, Slovakia, Scotland, Wales, the Czech Republic and Denmark (>10%) and lower in Iceland, Norway, Italy, Belgium Flemish, Israel and the Republic of North Macedonia (<4%).

**Table 4:** Early initiation in drunkenness (13 years old or younger) among adolescents aged 15 by country: percentage in each survey year, percentage of change between 2002^a^ and 2014, and PR (95% CI) of global comparison (2002–14)^a^ and 4-year period comparisons (2002–06, 2006–10 and 2010–14)

Country	2002 (%)	2006 (%)	2010 (%)	2014 (%)	Change 2002–14^a^ (%)	Global comparison (2002–14)^a^ PR (95% CI)	2002–06 PR (95% CI)	2006–10 PR (95% CI)	2010–14 PR (95% CI)
Austria	26.9	24.0	15.1	7.5	−72	0.277 (0.223–0.343)	0.891 (0.783–1.015)	0.628 (0.543–0.727)	0.494 (0.394–0.619)
Belgium (Flemish)	14.7	11.2	9.8	3.8	−74	0.260 (0.200–0.337)	0.761 (0.640–0.906)	0.880 (0.706–1.096)	0.388 (0.289–0.520)
Belgium (French)	16.4	14.0	10.2	4.8	−71	0.292 (0.231–0.368)	0.849 (0.711–1.015)	0.732 (0.595–0.901)	0.469 (0.363–0.606)
Canada	18.6	16.2	16.5	9.9	−47	0.476 (0.398–0.570)	0.868 (0.746–1.009)	0.987 (0.869–1.122)	0.556 (0.473–0.653)
Croatia	16.0	18.0	16.9	11.4	−29	0.715 (0.600–0.852)	1.123 (0.957–1.317)	0.942 (0.820–1.082)	0.676 (0.578–0.790)
Czech Republic	20.4	18.2	17.6	10.7	−48	0.524 (0.444–0.619)	0.890 (0.773–1.024)	0.968 (0.832–1.126)	0.609 (0.511–0.726)
Denmark	31.0	20.2	20.9	10.4	−66	0.334 (0.279–0.401)	0.652 (0.573–0.741)	1.037 (0.891–1.206)	0.495 (0.406–0.603)
England	30.7	23.6	16.7	9.4	−69	0.308 (0.260–0.365)	0.774 (0.688–0.872)	0.804 (0.681–0.949)	0.495 (0.403–0.607)
Estonia	24.2	27.9	23.8	18.9	−22	0.784 (0.674–0.911)	1.156 (1.019–1.311)	0.852 (0.753–0.964)	0.796 (0.686–0.923)
Finland	37.7	22.6	18.7	12.9	−66	0.341 (0.298–0.390)	0.599 (0.536–0.670)	0.826 (0.727–0.939)	0.690 (0.595–0.799)
France	9.8	–	9.3	6.3	−36	0.659 (0.525–0.828)	–	–	0.698 (0.548–0.888)
Germany	16.7	11.2	8.7	8.0	−52	0.485 (0.404–0.582)	0.673 (0.576–0.786)	0.782 (0.643–0.952)	0.922 (0.741–1.147)
Greece	7.4	6.6	6.4	6.1	−18	0.829 (0.621–1.106)	0.911 (0.686–1.210)	0.950 (0.714–1.264)	0.958 (0.717–1.280)
Hungary	9.2	11.9	12.5	9.8	+7	1.066 (0.830–1.369)	1.299 (1.027–1.643)	1.090 (0.886–1.341)	0.753 (0.601–0.942)
Iceland	–	9.2	6.6	2.3	−75	0.246 (0.189–0.322)	–	0.722 (0.598–0.871)	0.341 (0.264–0.441)
Ireland	15.9	17.6	13.9	5.5	−65	0.343 (0.265–0.444)	1.106 (0.922–1.327)	0.788 (0.672–0.926)	0.393 (0.308–0.501)
Israel	5.6	–	7.0	3.8	−32	0.685 (0.484–0.969)	–	–	0.554 (0.397–0.771)
Italy	5.2	4.2	5.4	3.3	−37	0.640 (0.436–0.941)	0.807 (0.564–1.155)	1.288 (0.915–1.813)	0.616 (0.426–0.892)
Latvia	14.0	21.3	25.7	12.9	−8	0.920 (0.755–1.122)	1.517 (1.257–1.831)	1.208 (1.048–1.393)	0.502 (0.430–0.586)
Lithuania	26.3	24.0	30.1	19.8	−25	0.755 (0.669–0.854)	0.915 (0.819–1.023)	1.251 (1.122–1.394)	0.660 (0.586–0.744)
Luxembourg	–	11.3	7.3	4.8	–58	0.426 (0.315–0.577)	–	0.644 (0.508–0.816)	0.662 (0.477–0.919)
Malta	7.2	10.2	–	8.9	+24	1.231 (0.848–1.788)	1.405 (0.923–2.137)	–	–
Netherlands	11.3	11.8	5.7	4.4	−61	0.383 (0.283–0.517)	1.045 (0.845–1.292)	0.474 (0.365–0.616)	0.773 (0.551–1.084)
Norway	17.0	9.2	6.2	3.2	−81	0.190 (0.130–0.276)	0.542 (0.447–0.657)	0.670 (0.514–0.873)	0.522 (0.345–0.791)
Poland	14.2	12.1	9.0	7.9	−44	0.559 (0.454–0.688)	0.854 (0.732–0.995)	0.747 (0.611–0.913)	0.877 (0.687–1.120)
Portugal	8.2	8.6	7.4	5.4	−34	0.666 (0.482–0.921)	1.050 (0.786–1.404)	0.860 (0.671–1.103)	0.737 (0.554–0.982)
Rep. North Macedonia	4.5	6.9	6.6	3.8	−16	0.840 (0.590–1.197)	1.509 (1.125–2.025)	0.960 (0.744–1.239)	0.580 (0.420–0.801)
Romania	–	12.6	28.7	9.7	−23	0.767 (0.624–0.943)	–	2.278 (1.961–2.647)	0.337 (0.283–0.401)
Russian Federation	14.6	19.1	12.5	4.2	−71	0.290 (0.221–0.380)	1.309 (1.156–1.482)	0.653 (0.560–0.761)	0.340 (0.255–0.452)
Scotland	30.2	22.3	21.9	11.0	−64	0.390 (0.328–0.462)	0.739 (0.657–0.832)	0.978 (0.878–1.089)	0.539 (0.457–0.635)
Slovakia	–	18.8	13.3	11.1	−41	0.590 (0.493–0.706)	–	0.709 (0.598–0.841)	0.833 (0.693–1.000)
Slovenia	16.5	14.2	17.3	8.4	−49	0.509 (0.410–0.630)	0.862 (0.715–1.039)	1.218 (1.037–1.430)	0.484 (0.400–0.587)
Spain	8.8	8.8	15.9	6.3	−28	0.712 (0.581–0.873)	0.985 (0.788–1.231)	1.835 (1.495–2.253)	0.394 (0.328–0.473)
Sweden	20.4	10.1	11.9	4.8	−76	0.233 (0.190–0.285)	0.495 (0.410–0.598)	1.180 (0.973–1.430)	0.398 (0.324–0.489)
Switzerland	10.5	12.3	10.0	5.3	−50	0.504 (0.400–0.635)	1.171 (0.958–1.432)	0.806 (0.669–0.972)	0.533 (0.428–0.664)
Ukraine	13.5	14.6	9.5	9.4	−30	0.672 (0.551–0.818)	1.040 (0.875–1.236)	0.654 (0.546–0.785)	0.987 (0.804–1.211)
Wales	32.7	22.7	19.5	10.7	−67	0.299 (0.244–0.366)	0.695 (0.611–0.791)	0.857 (0.745–0.986)	0.502 (0.407–0.619)

Greenland and USA are not included because they participated in only two rounds.

a For Iceland, Luxembourg, Romania, and Slovakia the change and the global comparison refer to 2006–14.

### Summary of results

The categorization of countries depending on their trends for each alcohol-related behaviour is shown in Supplementary Material B. Some crucial points can be highlighted here. In general, most countries showed decreases in all measures between 2002 and 2014 (independently of the timing and shape of trends). However, despite the overall decrease, there is variability both within and across countries. Concerning the timing, weekly drinking showed the higher number of countries starting to decline in 2002, followed by drunkenness. It should be also highlighted that a considerable number of countries started to decline in 2010. Regarding the pattern, only England and Wales presented a linear downward trend in all four variables since 2002 (followed by Finland with three). The rest of the countries decreasing since 2002 showed either a linear decrease in just one or two behaviours or presented periods of stability or increase. In contrast, some countries remained stable from 2002 to 2014 in one (Croatia, Czech Republic, Estonia, France, Israel, Italy, Latvia, Malta and Romania) or two variables (Hungary). Special attention needs to be paid to Greece and the Republic of North Macedonia, which showed stability between 2002 and 2014 in three measures. Of particular importance is countries showing increases (Croatia and Malta for drunkenness and Slovenia for early initiation in alcohol consumption). In relation to the magnitude, around half of the countries showed substantial reductions in the four measures with Iceland, Ireland, Norway and Sweden decreasing by 50% or more in all measures.

## Discussion

This article presents an overview of cross-national trends in adolescent alcohol consumption. Our study found a general decrease in all alcohol measures in most countries over the 12-year period. The results are consistent with the previous findings in Europe,^1^ USA,^3^ Australia,^14^ New Zealand^15^ and Japan^16^ reporting overall declines in adolescent drinking since the turn of the century. It should be highlighted that in some cases, more than half of countries have presented reductions of around 50%, reflecting a massive cultural shift in a relatively short period.

Despite the global decrease detected, this study adds a more detailed analysis at country level to identify specific trends, paying special attention to the beginning and pattern of trends. Regarding the timing, some variability between the four alcohol-related behaviours was found, showing weekly drinking the higher number of countries starting to decline in 2002. Our results are partially consistent with Vashishtha et al.,^5^ who found that the timing of trends in past-month drinking varied markedly across countries. However, they reported that only Northern Europe started in the early 2000s, followed by Western Europe in the mid-2000s and no discernible pattern in Eastern and Southern Europe. The higher number of countries decreasing in weekly use since 2002 compared with past-month drinking may suggest an earlier decrease in more regular drinking.

Focusing on the pattern of trends, our data showed that linear declines are scarce. On the contrary, most countries presented stability or increases over some parts of the time range. These findings are in line with Krauss et al.,^7^ who found decreasing concave trends when analysing weekly drinking by regions with ESPAD data. In our study, the country-level analysis enabled the identification of linear decreases in specific countries. However, we did not find the increasing concave trend they found in the Balkans (all countries reduced their weekly consumption levels between 2002 and 2014 except Israel, Republic of North Macedonia and Romania where no significant change was found). Note that in Kraus et al.^7^ countries were grouped by regions and a different measure of weekly drinking was used (having drunk alcohol in three occasions or more in the last 30 days), what may explain this different finding.

Concerning the magnitude of declines, most countries showed substantial reductions in all alcohol measures, what supports the central notion of Skog’s theory of collectivity of drinking cultures in which changes in alcohol use happen at all levels of consumption.^17^ However, some countries such as the Czech Republic, France, Greece, Hungary, Italia, Latvia and Spain reduced their levels of weekly drinking, but not, or to a lesser degree, of drunkenness. Croatia and Malta even increased the proportion of 15-year-olds who reported having been drunk twice or more in their lives. Most of these exceptions are Southern countries, which may indicate a higher acceptance of intoxication in countries traditionally less drunkenness-oriented.^6^ Likewise, despite decreasing their levels of drunkenness, high rates still exist in countries that have been successful in reducing overall levels of adolescent alcohol consumption (e.g. UK and Denmark) reinforcing the idea that heavy drinking is not necessarily most common in the same countries where alcohol consumption is most common.^18^ It is therefore likely that different factors are driving these trends, and further investigation of excessive drinking is warranted as this poses a particular risk to the developing adolescent and different preventive strategies are needed. Denmark is a particular case. Being usually included in the Nordic group, this country showed poorer outcomes than Iceland, Norway, Sweden and, to a lesser extent, Finland. A higher openness to freedom and self-determination in relation to alcohol in youth and families^19^ as well as in society^20^ may be behind these differences. Interestingly, different trends in the total alcohol per capita consumption were observed in these countries between the mid-90s and the first decade of the 21st century, decreasing in Denmark while increasing in the rest of the Nordic countries confirming the singularity of the case of Denmark.^21^ Special attention should be paid to those countries not decreasing in several behaviours. That is the case for Greece, the Republic of North Macedonia, Hungary, Croatia and Malta, all located in the southeast of Europe. For example, in Malta, the adverse outcomes found in our study coincided in time with a decrease in perceived risk of regular alcohol use and heavy episodic drinking.^22^ Weak enforcement of minimum legal age regulations or the lack of a national alcohol policy until recently^23^ may be among the factors involved.

There are some important highlights with respect to drinking patterns across geographical regions. First, it is difficult to confirm the existence of robust patterns by regions for any of the alcohol-related behaviours since our data revealed that not all countries within the same region showed similar results, but rather there was some variability. Nevertheless, certain particularities are worth mentioning. In line with Vashishtha et al.’s^5^ findings on past-month drinking, our study found greatest reductions in weekly use in Nordic and British Isles countries. Other countries showing steepest declines were Estonia and the Russian Federation. Weekly drinking remained higher in some South-Eastern countries (Malta, Italy, Croatia and Greece) while lower in Nordic countries (Iceland, Norway, Sweden and Finland) both in 2002 and 2014. In contrast, some Northern countries (England, Scotland, Wales, Denmark, Finland, Estonia and Lithuania) showed high drunkenness levels and early initiation in drunkenness both in 2002 and 2014. These findings are partially consistent with previous studies reporting higher rates of regular drinking in Southern countries and lower regular, but heavier, drinking in Northern countries.^6^^,^^7^ In this sense, in a study about drinking motives, Kuntsche et al.^24^ found that, in 14–16-year-olds, the strong positive link between enhancement motives and drinking frequency found in some Southern countries was significantly weaker in some Northern countries.

However, in 2014, Southern countries such as Portugal and Spain showed low levels of weekly use and countries from different regions showed high levels of drunkenness and/or early initiation in drunkenness (Croatia, Czech Republic, Hungary, Slovakia or Slovenia). In early initiation in alcohol consumption, even more variability between countries was found. In this respect, the variability in findings may be, to some extent, consonant with increasing homogenization of drinking cultures across countries proposed in previous studies.^9–11^ Gordon et al.^11^ suggested that this may reflect factors such as homogenization of lifestyles, urbanization, greater female independence, globalization of alcohol marketing and moves towards greater homogeneity of legislation and regulation. In sum, in the light of these results, conclusions as to the existence of robust drinking patterns across geographical regions should be treated cautiously.

Further research is needed in order to clarify and understand the factors associated with adolescent drinking. Previous studies suggested factors related to adolescents, their families and society.^2^^,^^25–27^ The most robust evidence seems to be associated with shifts in parental practices^27^ and in unorganized leisure time with peers,^28^ whereas the widespread assumption relating the rise of new technologies with reductions in adolescent drinking has not been supported by research.^2^^,^^28^ Regarding alcohol control policies (i.e. imposing a minimum legal drinking age, restricting availability, regulating advertising, providing information and education), there is a lack of strong agreement on their effects on alcohol-related behaviours.^29–33^ However, changes in affordability (i.e. taxation and price regulation) have stood out as one of the most effective measures.^29^^,^^34^^,^^35^ Efforts should be focused not just on what may be related to the decrease, but also on exploring why the decline is present only (or to a larger extent) in some groups of adolescents and not others. In this sense, some studies have detected inequalities in trends in alcohol consumption regarding sex, socioeconomic status or native/immigrant.^35–38^ A recent study conducted with HBSC data showed that, despite the confirmed overall downward trend in adolescent alcohol use between 2002 and 2014, socioeconomic inequalities related to family material affluence (higher lifetime use, weekly drinking and drunkenness among adolescents from a higher position) and perceived family wealth (higher drunkenness among adolescents who perceived their families to be poor) persisted in lifetime and weekly use and even increased in drunkenness.^35^ At regional- and country-level, future studies should analyze the reasons why specific countries have not decreased over time, or even while decreasing in some behaviours, have remained among the most prevalent in others. High risk (excessive) drinkers are now a minority in most countries but likely that high risk drinking associated with multiple other risk factors, both social and behavioural, so should be a particular target for preventive action. It is also important to examine why some policies work in some countries, but not in others. Some authors state that future research on this area needs to widen the perspective and frame the analyses within a historical and generational perspective in which changes in alcohol-related behaviours are part of a larger shift in the way adolescents are, think and behave.^7^^,^^25^

There are some limitations to our study. First, those associated with the use of self-reported measures; second, the lack of data for some countries in specific measures and years. We only analyzed data up to 2013/14 because of changes to the HBSC mandatory questionnaire that meant that not all of the variables were included in all countries in the most recent (2017/18) survey round. Third, response rates by country and year are not available. However, for 2010 and 2014, the rates were reported to be higher than 60% in most countries.^39^^,^^40^ Even with high response rates, it is possible for samples to be biased and therefore vary between years in ways that might impact on the results. All our analyses were able to account for some level of response bias via the inclusion of national-level post-stratification weights provided. Finally, the statistical analysis did not allow for variance partitioning, so the model used cannot simultaneously distinguish the different variance components (i.e. time and place). However, this study presents important strengths, such as the large sample and number of countries, the use of a cross-validated questionnaire that allows for comparisons and the inclusion of four different measures to assess alcohol consumption. All these strengths make this study a comprehensive resource to better understand the evolution of adolescent alcohol consumption across countries and regions that can enable policy-makers to take action to tackle this major public health concern.

In conclusion, there has been a general decline in adolescent alcohol-related behaviours across Europe and North America between 2002 and 2014. However, variability both within a country (depending on the alcohol-related behaviour under study) and across countries (in the beginning and shape of trends) has been observed, with some countries showing less optimal results. Although some particularities have persisted over time, the existence of robust patterns by regions is unclear.

## Supplementary data

Supplementary data are available at *EURPUB* online.

## Supplementary Material

ckab024_Supplementary_Data
