# Supplementary material for: Cross-national time trends in adolescent alcohol use from 2002 to 2014
Source: Eur J Public Health. 2021 Jul 14;31(4):859–66. doi: 10.1093/eurpub/ckab024 (PMC8790077; doi:10.1093/eurpub/ckab024)
Supplement: ckab024_Supplementary_Data [file ckab024_supplementary_data.zip › ckab024-suppl_data/updated- Supplementary materials.docx]

Supplementary material A. Sample of the study per country, year of the study, and sex.

|  | 2002 | | 2006 | | 2010 | | 2014 | | TOTAL |
| --- | --- | --- | --- | --- | --- | --- | --- | --- | --- |
|  | Boys | Girls | Boys | Girls | Boys | Girls | Boys | Girls |  |
| Austria | 643 | 634 | 693 | 801 | 885 | 935 | 545 | 719 | 5855 |
| Belgium (Flemish) | 1027 | 1003 | 831 | 785 | 680 | 546 | 1021 | 696 | 6589 |
| Belgium (French) | 631 | 750 | 731 | 683 | 669 | 672 | 924 | 1008 | 6068 |
| Canada | 528 | 679 | 1095 | 1194 | 2685 | 2756 | 2506 | 2467 | 13910 |
| Croatia | 619 | 816 | 773 | 857 | 1197 | 1227 | 1031 | 915 | 7435 |
| Czech Republic | 806 | 854 | 842 | 823 | 747 | 775 | 852 | 908 | 6607 |
| Denmark | 656 | 713 | 762 | 790 | 577 | 649 | 584 | 679 | 5410 |
| England | 801 | 963 | 709 | 742 | 494 | 624 | 816 | 792 | 5941 |
| Estonia | 619 | 648 | 801 | 786 | 661 | 737 | 638 | 631 | 5521 |
| Finland | 867 | 874 | 790 | 895 | 1008 | 1102 | 956 | 1009 | 7501 |
| France | 1301 | 1313 | 1139 | 1083 | 904 | 1002 | 865 | 875 | 8482 |
| Germany | 842 | 899 | 1271 | 1281 | 736 | 904 | 1061 | 1043 | 8037 |
| Greece | 643 | 681 | 650 | 766 | 842 | 806 | 638 | 682 | 5708 |
| Greenland | 100 | 138 | 207 | 210 | 189 | 208 | 157 | 163 | 1372 |
| Hungary | 498 | 812 | 550 | 637 | 799 | 934 | 535 | 565 | 5330 |
| Iceland | - | - | 947 | 936 | 1873 | 1807 | 1659 | 1657 | 8879 |
| Ireland | 345 | 574 | 914 | 771 | 962 | 733 | 576 | 944 | 5819 |
| Israel | 697 | 850 | 758 | 1239 | 681 | 671 | 857 | 1007 | 6760 |
| Italy | 541 | 679 | 678 | 657 | 764 | 782 | 640 | 622 | 5363 |
| Latvia | 481 | 631 | 628 | 702 | 666 | 709 | 784 | 942 | 5543 |
| Lithuania | 981 | 923 | 940 | 921 | 945 | 847 | 904 | 794 | 7255 |
| Luxembourg | - | - | 776 | 731 | 702 | 680 | 499 | 580 | 3968 |
| Malta | 310 | 349 | 184 | 170 | 0 | 0 | 318 | 327 | 1658 |
| Netherlands | 637 | 636 | 672 | 691 | 730 | 727 | 658 | 699 | 5450 |
| Norway | 799 | 823 | 818 | 716 | 711 | 628 | 467 | 503 | 5465 |
| Poland | 1022 | 1105 | 1092 | 1195 | 685 | 725 | 700 | 784 | 7308 |
| Portugal | 378 | 422 | 613 | 770 | 680 | 873 | 630 | 730 | 5096 |
| Rep. North Macedonia | 672 | 727 | 952 | 944 | 814 | 722 | 742 | 715 | 6288 |
| Romania | - | - | 606 | 999 | 1046 | 956 | 633 | 809 | 5049 |
| Russia | 1138 | 1436 | 1238 | 1516 | 919 | 928 | 659 | 786 | 8620 |
| Scotland | 578 | 571 | 1108 | 1090 | 1232 | 1335 | 951 | 918 | 7783 |
| Slovakia | - | - | 591 | 661 | 961 | 953 | 971 | 864 | 5001 |
| Slovenia | 543 | 509 | 780 | 781 | 914 | 901 | 744 | 871 | 6043 |
| Spain | 821 | 935 | 1519 | 1546 | 962 | 1041 | 1761 | 1998 | 10583 |
| Sweden | 609 | 609 | 752 | 774 | 1059 | 1031 | 1358 | 1408 | 7600 |
| Switzerland | 770 | 731 | 733 | 767 | 1138 | 1108 | 1100 | 1112 | 7459 |
| Ukraine | 730 | 871 | 835 | 994 | 881 | 1016 | 792 | 902 | 7021 |
| USA | 754 | 871 | 649 | 635 | 968 | 924 | - | - | 4801 |
| Wales | 603 | 561 | 675 | 675 | 855 | 782 | 729 | 703 | 5583 |
| TOTAL | 23990 | 26590 | 31302 | 33214 | 34221 | 34756 | 32261 | 33827 | 250161 |

Supplementary material B. Categorization of countries by their trends in each of the four alcohol-related behaviours.

|  | *Weekly consumption*  (Drinking alcohol at least weekly) | *Drunkenness*  (Been drunk two times or more in lifetime) | *Early initiation in alcohol consumption*  (13 years old or younger) | *Early initiation in drunkenness*  (13 years old or younger) |
| --- | --- | --- | --- | --- |
| Decrease from 2002 | **Belgium (Flemish), Belgium (French),Canada, Denmark, England, Estonia,** Finland^e^, Germany^c^, Greece^d^, Greenland^c,e^, Hungary^c,e^, Lithuania^d^, **Netherlands**, Norway^c^, Poland^c^, Portugal^e^, Russian Federation^e^, **Scotland**, Sweden^c^, Switzerland^c^, USA^c^, **Wales**. | Belgium (Flemish) ^c^, Canada^c^, Denmark^c^, **England, Finland**, Germany^c^, Greenland^c^, Norway^c^, Scotland^c^, Slovania^d^, Sweden^c^, Switerzland^c^, **Ukraine, USA, Wales**. | **Austria, Belgium (Flemish),** Canada^c^, **Czech Republic**, Denmark^c^, **England**, **Finland**, **Germany**, Lithuania^c^, **Norway**, **Scotland**, Sweden^c^, **Wales**. | Belgium (Flemish) ^c^, Denmark^c^, **England**, **Finland**, Germany^e^, **Norway**, Poland^e^, Scotland^c^, Sweden^c^, **Wales**. |
| Decrease from 2006 | Iceland, Ireland, Italy, Latvia, Rep. North Macedonia^a,e^, Ukraine. | Croatia^b,e^, Estonia, Iceland, Ireland, Italy^e^, Luxembourg, Netherlands^e^, Poland, Rep. North Macedonia^a,e^, Russian Federation. | Belgium (French), Hungary, Iceland, Ireland, Luxembourg^e^, Netherlands, Poland, Portugal, Romania^e^, Russian Federation, Slovakia, Switzerland, Ukraine^e^. | Austria, Belgium (French), Estonia, Iceland, Ireland, Luxembourg, Netherlands^e^, Russian Federation, Slovakia^e^, Switzerland, Ukraine^e^. |
| Decrease from 2010 | Austria, Croatia, Czech Republic, France, Luxembourg, Romania^a^, Slovakia, Slovenia, Spain. | Austria, Belgium (French), Czech Republic^a^, France^a^, Israel, Latvia, Lithuania, Portugal, Romania, Slovakia, Spain. | Croatia^a^, Estonia^a^, Israel, Italy^a^, Latvia, Rep. North Macedonia, Slovenia^b^, Spain. | Canada, Croatia, Czech Republic, France, Hungary^a^, Israel, Italy, Latvia^a^, Lithuania, Portugal, Rep. North Macedonia ^a^, Romania, Slovenia, Spain. |
| Other trends | Israel^a^, Malta. | Greece^a^, Hungary^a^, Malta^b^. | Greece^a^ Malta. | Greece^a^, Malta ^a^. |

^a^ Stability between 2002 and 2014; ^b^ Increase between 2002 and 2014; ^c^ Stability between 2006 and 2010; ^d^ Increase between 2006 and 2010; ^e^ Stability between 2010 and 2014.

Countries with significant decreases since 2002 are denoted in bold.
